# 

**DOI:** 10.1192/bjb.2019.38

**Published:** 2019-08

**Authors:** Suhanthini Farrell

**Affiliations:** Consultant Psychiatrist, Lowry Unit, Prestwich Hospital, Greater Manchester Mental Health NHS Foundation Trust, Manchester, UK. Email: suhanthini.farrell@gmmh.nhs.uk

How can we understand psychosis without experiencing it ourselves? How can we convey such a complex, bizarre and (as Jaspers would have it) *un-understandable* experience to others, particularly those new to psychiatry? It is a difficult task.

Karin Fossum, the respected Norwegian crime writer, does not shy away from confronting the more unpalatable aspects of the human mind. Her portfolio has included an exploration of the psychological motivation of paedophiles, elder abusers, and vulnerable people who are drawn into crime. Her focus is not on what, but why.

*The Whisperer* centres on the unremarkable, quiet, middle-aged Ragna Reigel, who, after surgery, was left with a disability that means she cannot talk above a hoarse whisper. Isolated and ignored, no-one notices (or particularly cares) when her thoughts and behaviour start to change, until it is rather too late.

Fossum has used her talent for drawing us into the minds of the misunderstood to powerful effect. Through the format of a sensitively conducted police interview, we are carried alongside Reigel as she develops psychosis. It is an unsettling and disorienting experience.

The book captures the sense of unease and apprehension that come with delusional mood. This progresses into more defined delusions and paranoia that permeate every aspect of Reigel's life. The loss of her sense of agency and experience of perplexity and distorted perceptions is well described.

‘…her overview was slipping…her body had been knocked out of its natural measured rhythm…life was no longer safe…external powers were taking over.’

‘The night was no longer silent, she could hear the seven billion people who lived on this earth. They were breathing like an enormous beast, cackling and screaming and wailing.’

The language changes subtly as Reigel deteriorates, reflecting her increasingly disordered thoughts as she struggles to make sense of what is happening to her.

Through Reigel's isolation and loneliness, the book raises broader questions about how we as a society care for the vulnerable and marginalised. The effect of disability and impaired communication on mental health is considered, as is the humane treatment of offenders with mental disorders.

The book's great strength is its accessibility. It can be enjoyed on its own merits as a work of fiction; yet it also communicates an experience of psychosis in a way no textbook can. I would recommend it to medical students and trainee psychiatrists, but even seasoned psychiatrists could gain a great deal from it. Empathy starts with understanding the experience of those we care for, and *The Whisperer* – quietly, unassumingly and unnervingly – helps us to do just that.

